# A quality evaluation strategy for residual host cell proteins based on orthogonal analysis

**DOI:** 10.3389/fbioe.2026.1886078

**Published:** 2026-07-14

**Authors:** Xinyue Hu, Ping Lyu, Dan Wang, Yi Li, Kezheng Xu, Feng Ling, Minhua Dou, Chenggang Liang, Jing Li

**Affiliations:** 1 National Institutes for Food and Drug Control, Beijing, China; 2 Huzhou Institute of Applied Technology, Chinese Academy of Sciences, Zhejiang, China

**Keywords:** adjacent dilution-gradient back-calculation recovery, antibody coverage, ELISA, host cell proteins (HCPs), IMBS-MS/MS

## Abstract

Accurate quantification of host cell proteins (HCPs) is essential for the safety and quality control of biopharmaceuticals. Although enzyme-linked immunosorbent assay (ELISA) remains the current industry gold standard, heterogeneity among the polyclonal antibody repertoires of different commercial kits often results in substantial method-dependent bias. This study aimed to establish a novel strategy for the quality evaluation of HCP detection kits for biologics. Two Chinese hamster ovary cell-derived recombinant protein drug substances with different HCP burdens were utilized as model samples to functionally compare nine mainstream commercial kits. A quantitative metric, adjacent dilution-gradient back-calculation recovery, was introduced to comprehensively assess dilution linearity, sensitivity, and resistance to matrix interference. To eliminate evaluation bias, an unsupervised machine learning workflow utilizing hierarchical clustering analysis (HCA) based on a 10-dimensional functional feature matrix was implemented for objective kit stratification. Functional assessment showed clear stratification among the nine kits. Kit A displayed the most robust analytical performance, with adjacent dilution-gradient back-calculation recovery strictly maintained within 80%–120% over dilution windows ranging from 16-fold to 128-fold, thereby overcoming the hook effect and false-negative risks, as mathematically validated by its unique branching under the HCA model. IMBS-MS/MS analysis demonstrated that although the mainstream kits evaluated (A and I) both achieved overall antibody coverage above 80%, only Kit A showed notable concordance between high coverage and superior functional performance. High antibody coverage is a necessary but insufficient condition for dependable ELISA performance. Pursuit of overall coverage alone cannot comprehensively reflect the quantitative reliability of a kit. Therefore, we recommend an orthogonal evaluation framework that combines functional verification, with priority given to robust dilution linearity, coupled with HCP coverage analysis during bioprocess development and quality control, to ensure scientifically sound and compliant impurity monitoring.

## 1 Introduction

The production of recombinant therapeutic proteins utilizing mammalian cell lines, particularly Chinese hamster ovary (CHO) cells, has become a main platform of the modern biopharmaceutical industry for products such as monoclonal antibodies, Fc fusion proteins, and enzyme replacement therapies. However, host cells inevitably release large quantities of endogenous proteins, collectively referred to as host cell proteins (HCPs), throughout the upstream culture and downstream purification processes. HCPs may originate from endogenous host cell proteins, apoptosis, metabolites, or cell lysis. Although these proteins play significant roles in normal cellular physiology, they may be co-purified with the target molecule during manufacturing (Stollar and Smith, 2020). Under physiological metabolism, host cells also secrete proteins into the culture medium; such secreted proteins may be purified together with the product and continue as impurities in the final drug substance. Although downstream purification steps, such as Protein A affinity chromatography, ion-exchange chromatography, and hydrophobic interaction chromatography, are designed to efficiently remove impurities, trace HCPs may still co-elute and remain in the final product. Residual HCPs constitute a crucial class of process-related impurities, and their potential risks are multifaceted. From the perspective of physicochemical stability, certain HCPs possess enzymatic activity, including protease, lipase, phosphatase, and glycosidase activities, and can catalyze the degradation, deamidation, oxidation, or excipient hydrolysis (e.g., polysorbate 20/80) of proteins, thereby inducing protein aggregation, visible particles, or loss of biological activity. From the perspective of immunogenicity, HCPs, as exogenous non-human proteins, may be identified as antigens by the immune system of the patient and induce anti-drug antibodies (ADAs), which may attenuate efficacy and trigger cross-reactivity, hypersensitivity, or even life-threatening immunotoxic events (Wang et al., 2022; Ito et al., 2024; Yao et al., 2025). From the perspective of regulatory compliance, major regulatory authorities regard residual HCP levels as a critical quality attribute (CQA) and require stringent monitoring and control throughout the life cycle of the product, from process development to commercial manufacturing (Lu et al., 2023). Consequently, control and risk assessment of HCPs are of vital importance across the biopharmaceutical industry. Nonetheless, these tasks remain highly challenging because HCPs are heterogeneous in immunogenic potential, biological activity, and biochemical properties—all of which may influence co-purification with the therapeutic product, including the so-called “hitchhiker phenomenon.”

At present, enzyme-linked immunosorbent assay (ELISA) is widely accepted as the industry-standard method for HCP quantification because of its high sensitivity, high throughput, operational simplicity, and ease of integration into GMP quality systems (Van Manen-Brush et al., 2020; Pilely et al., 2022; Giordano et al., 2024). However, the reliability of ELISA is highly dependent on the quality of its core reagent, namely, polyclonal anti-HCP antibodies. Ideally, such antibodies should provide broad and balanced immunorecognition of the complex HCP mixtures that may arise during manufacturing; that is, they should display high antibody coverage (Seisenberger et al., 2023; Pilely et al., 2022). In principle, the antibodies should recognize complex HCP mixtures broadly and evenly throughout the process (Pilely et al., 2020; Graf et al., 2021). In practice, however, the situation is far more complex. Commercial ELISA kits differ substantially in immunogen source, such as specific CHO sublines, culture modes, harvest time points, and cellular lysis components, as well as in animal immunization strategy, including species used, number of immunizations, adjuvant type, and in antibody purification procedures, such as antigen-affinity purification and removal of highly abundant cross-reactive proteins. These differences generate pronounced heterogeneity in epitope recognition spectra, affinity distributions, and cross-reactivity among antibody repertoires (Takagi et al., 2022). As a result, measurements of the same process sample may differ by several-fold or even by an order of magnitude across kits, and linear dynamic range, matrix effect, and lower detection limit may also vary substantially. Such method-dependent bias severely constrains the reliability of impurity-control strategies during process characterization, technology transfer, and regulatory submission. To address these challenges, both the European Pharmacopoeia (EP 2.6.34) and the United States Pharmacopeia (USP <1132>) have established explicit expectations for HCP testing. Specifically, the USP emphasizes that method validation should include evaluation of sample linearity and antibody coverage and recommends the utilization of orthogonal methods, such as mass spectrometry (USP 1132.1), for supplementary confirmation (Seisenberger et al., 2022).

The reliability of ELISA depends on the antibody coverage of polyclonal anti-HCP antibodies, namely, the breadth and efficiency with which they recognize HCPs in the sample. In recent years, rapid advances in proteomic technologies, especially liquid chromatography-tandem mass spectrometry (LC-MS/MS), have provided powerful tools for resolving HCP composition, identifying difficult-to-clear specific HCP impurities, and objectively assessing ELISA antibody coverage. Among these, immunoaffinity capture-MS (IAC-MS) has been extensively adopted (Waldera-Lupa et al., 2021). In IAC-MS, HCPs recognized by the antibody are first captured and then identified by mass spectrometry, allowing direct analysis of the antibody’s ability to recognize HCPs present in real samples. This strategy overcomes the limitations of traditional methods, such as two-dimensional Western blotting (King et al., 2024), and provides a more thorough picture of coverage. For example, one study employed isobaric-label affinity purification-MS (AP-MS) to assess the immunoreactivity of eight commercial anti-HCP antibodies raised in different host animals and identified 34 abundant but poorly recovered proteins that could otherwise be underestimated in biopharmaceutical quality assessments (Takagi et al., 2022). Immunomagnetic bead separation-LC-MS/MS (IMBS-LC-MS/MS) utilizes magnetic microspheres with covalently coupled antibodies to efficiently capture HCPs from complex samples through antigen-antibody recognition, followed by LC-MS/MS-based identification and semi-quantitative evaluation of the captured proteins. This approach is operationally convenient, displays low nonspecific adsorption, requires minimal sample, and is readily automated. The large surface area and suspended-liquid characteristics of magnetic microspheres noticeably improve antigen–antibody binding kinetics, whereas magnetic separation avoids the protein loss and structural disruption that may occur during centrifugation. The method is therefore particularly well suited for enrichment and identification of low-abundance HCPs and can offer a reliable proteomic basis for performance verification of ELISA methods.

With the rapid expansion of the biopharmaceutical industry and the continuous emergence of domestic kits, a common unresolved challenge is how to systematically and objectively evaluate and select HCP ELISA kits suitable for a given product in accordance with international regulations and sound scientific principles. The current study was designed to address this practical gap. Guided by current regulatory expectations, we employed two CHO cell-derived recombinant protein drug substances as model samples to systematically compare the performance of nine CHO HCP ELISA kits from a diverse range of manufacturers, with specific emphasis on key analytical attributes, such as linear range, precision, and accuracy. Furthermore, the detection capability of the selected kits was thoroughly evaluated from both immunorecognition and proteomic perspectives, and antibody coverage was experimentally characterized at the proteomic level. By jointly assessing the functional performance and immunorecognition range, this study provides substantial data support and an operational methodological framework for kit selection in the biopharmaceutical industry, as well as a technical foundation for improving ELISA kit quality and promoting the application of the kit with precise quality control of biological products.

## 2 Reagents and instruments

The IMBS-HCP immunoaffinity capture kit (Huzhou Shenke Biotechnology Co., Ltd., China, 01; containing NHS magnetic beads); Pierce™ Coomassie (Bradford) Protein Assay Kit (Thermo, Cat. #: 23200); Pierce™ Quantitative Colorimetric Peptide Assay (Thermo, Cat. #: 23275); sodium deoxycholate (SDC; Merck, Cat. #: 30970-25G); chloroacetamide (CAA; Merck, Cat. #: C0267-100G); tris(2-carboxyethyl)phosphine hydrochloride (TCEP; Sigma); SOLAμ™ SPE plate (Thermo, Cat. #: 77720, Cat. #: 60209-001); Monospin C18 desalting column (Shimadzu, Cat. #: 5010-21701); chromatographic column (Waters, Cat. #: 0476340181); and acetonitrile (Fisher, Cat. #: A955-4-4L) were used. The selection of the nine commercial CHO HCP ELISA kits (A–I) was guided by market prevalence in global and Chinese biopharmaceutical quality control, antibody host background (rabbit, sheep, goat or mixed polyclonals), and assay architecture. Specifically, the cohort covers both one-step incubation methodologies (Kits B, C, D, E, and H) and three-step incubation methodologies (Kits A, F, G, and I) to comprehensively evaluate process suitability. Nine CHO HCP ELISA kits were evaluated and designated A–I: A (rabbit polyclonal antibody, China), B (sheep polyclonal antibody, China), C (sheep polyclonal antibody, USA), D (goat polyclonal antibody, China), E (mixed rabbit/sheep polyclonal antibody, China), F (sheep polyclonal antibody, China), G (rabbit polyclonal antibody, China), H (sheep polyclonal antibody, China), and I (mixed rabbit/sheep polyclonal antibody, China). Recombinant protein drug substance 1 was alteplase (CHO cell-derived, 2.5 mg/mL, Takeda), for which the manufacturer utilized an in-house ELISA kit for release testing, with a certificate-of-analysis (COA) value of 450–750 ng/mg. Recombinant protein drug substance 2 was dulaglutide drug substance (CHO-K1 cell-derived, 30 mg/mL, Shandong Boan Biotechnology Co., Ltd.), for which the manufacturer utilized commercial kit C for release testing, with a mean COA value of approximately 20 ng/mg. Additional instruments included a microplate reader (BioTek), a Vanquish liquid chromatography system and Orbitrap Exploris 480 mass spectrometer (Thermo Fisher Scientific, USA), a magnetic rack (Beaver), an analytical balance (Mettler Toledo, Switzerland), and Proteome Discoverer software (Thermo Fisher Scientific, USA).

## 3 Experimental methods

### 3.1 Assessment of sample linearity

Sample measurements were assessed for linearity across different dilution factors. Drug substance 1 was tested over a 4-fold to 512-fold dilution range using 2-fold serial dilutions. Drug substance 2 was tested over a 5-fold to 320-fold dilution range using 2-fold serial dilutions. Residual HCPs were measured according to the instructions for each kit utilizing the following general procedure: 1) Samples, standards, and enzyme-labeled antibodies were incubated in duplicate wells; 2) plates were washed with wash buffer; 3) TMB substrate was added for color development; 4) the reaction was stopped; 5) optical density (OD) was measured with a microplate reader; and 6) data were processed by fitting a four-parameter logistic curve using calibrator concentration as the x-axis and OD as the y-axis, from which mean concentrations and coefficients of variation (CVs) were calculated.

### 3.2 Kit validation

#### 3.2.1 Linearity validation

Standard concentration was plotted on the x-axis, and OD was plotted on the y-axis for curve fitting. The valid analytical range was determined on the basis of accuracy, precision, and linearity results. The Limit of Quantification (LOQ) was individually established for each of the nine kits, defined strictly as the lowest concentration point on its respective linear standard curve that met the acceptance criteria for accuracy and precision.

#### 3.2.2 Precision validation

For within-run precision (repeatability) evaluation, drug substance 1 was diluted 128-fold and prepared in six parallel independent sample preparations (including separate dilution and handling steps, rather than replicate wells from a single tube) to calculate the CV within a single analytical run. For between-run (intermediate) precision evaluation, a 16-fold dilution of drug substance 1–representing the critical Minimum Required Dilution (MRD) threshold–was analyzed across three independent analytical days (one run per day).

#### 3.2.3 Accuracy validation

1. HCP standards at 810, 90, and 3.3 ng/mL were utilized as high-, medium-, and low-concentration samples, respectively, and the recovery and CV were calculated.
2. To assess the accuracy of the HCP method in drug substance samples, the standard was spiked into appropriate samples to demonstrate accurate spike recovery within the analytical range. Matrix interference may arise from buffer components and product matrix; therefore, the minimum dilution required to achieve acceptable spike recovery, typically 70%–130%, should be established for each sample type. For three batches of drug substance 1, 162 ng of standard was spiked across a 16-fold to 256-fold dilution series. For example, each batch was diluted 128-fold, and 400 μL of diluted drug substance 1 was mixed with 100 μL of the 810 ng/mL standard to prepare a spiked sample with a theoretical standard concentration of 162 ng/mL, after which spike recovery was calculated.

### 3.3 UPLC-MS/MS determination of antibody coverage

#### 3.3.1 Enrichment of HCPs

HCPs were enriched by using the IMBS-HCP immunoaffinity capture kit. Briefly, NHS magnetic beads were washed and coupled with 1 mg of the target antibody by rotary incubation at 25 °C for 2 h. After coupling, the beads were blocked with blocking solution. The antibody-coupled beads were then incubated with a sample solution containing 4 mg of HCP at room temperature for 2 h to allow specific binding. The beads were then washed stringently with wash solution, equilibration solution, and ultrapure water to remove nonspecifically adsorbed material. HCPs bound to the beads were stepwise-eluted utilizing IMBS elution solutions A and B. The eluates were combined and neutralized with neutralization buffer. Finally, the eluate was concentrated to a suitable volume using a 3-kDa ultrafiltration device, and the post-elution HCP concentration was determined using the Bradford assay. The above procedure was repeated until approximately 300 μg of HCP was obtained.

#### 3.3.2 Enzymatic digestion of HCPs

A total of 300 μg each of unenriched HCP sample and IMBS-enriched HCP sample was placed into separate 3-kDa ultrafiltration devices and buffer-exchanged with 0.1 M Tris, pH 8.0, to a final volume of 200 μL. Reduction and alkylation were then conducted by sequential addition to final concentrations of 1% SDC, 0.04 M CAA, and 0.01 M TCEP, followed by heating at 95 °C for 10 min. After cooling to room temperature, trypsin was added at an enzyme-to-protein ratio of 1:25 (w/w), and digestion was performed for 17 h at 37 °C. Formic acid was then added to a final concentration of 1% to stop the reaction. After low-temperature, high-speed centrifugation to remove precipitates, the supernatant was desalted, and the peptides were eluted with 60% acetonitrile containing 0.1% formic acid. The eluate was dried under nitrogen, reconstituted in 0.1% formic acid in water, and quantified using a peptide assay kit. For routine LC-MS analysis, 120 μg peptide was injected.

#### 3.3.3 LC-MS/MS acquisition

An ACQUITY UPLC peptide CSH C18 column (2.1 × 150 mm, 1.7 μm) was employed, with 0.1% formic acid in water (A) and 0.1% formic acid in acetonitrile (B) as the mobile phases. Gradient elution was programmed as follows: 0–5 min, 3% B; 5–7 min, 3% B; 7–15 min, 3%–8% B; 15–130 min, 8%–30% B; 130–154 min, 30%–55% B; 154–155 min, 55%–90% B; 155–160 min, 90% B; 160–160.1 min, 90%–3% B. The flow rate was 0.2 mL/min from 0 to 5 min, decreased from 0.2 to 0.1 mL/min from 5 to 160 min, and increased from 0.1 to 0.2 mL/min from 160 to 160.1 min. Column temperature was maintained at 60 °C, and the injection volume was 20 μL. Data were acquired in data-dependent acquisition mode with a spray voltage of 3.5 kV in positive-ion mode over an m/z range of 150–2000. Full-scan resolution was 60,000 at m/z 200, and MS/MS resolution was 15,000 at m/z 200.

#### 3.3.4 LC-MS/MS data processing and calculation

Database searching was conducted with Proteome Discoverer 3.1 against the UniProt Chinese hamster (*Cricetulus griseus*) reference proteome (Proteome ID: UP000001075, file version 20251104). The search parameters were as follows: fixed modification, carbamidomethyl (C); variable modifications, oxidation (M), acetyl (protein N-terminus), Met-loss (M), and Met-loss + acetyl (M); enzyme, trypsin; maximum missed cleavages, 2; precursor mass tolerance, 10 ppm; fragment mass tolerance, 0.02 Da. For data processing and coverage calculation, antibody coverage was calculated utilizing proteins supported by at least two unique peptides. Based on the database searching, we allowed C to denote the number of proteins common to both samples, and A and B denoted the numbers unique to each sample. Coverage was calculated employing the following equation:

$$\text{Coverage }\left(\%\right)=\left(A+C\right)/\left(A+B+C\right)\times 100\%,$$

where A is the number of proteins unique to the enriched HCP sample; B is the number of proteins unique to the unenriched HCP sample; and C is number of proteins common to both samples.

### 3.4 Unsupervised machine learning for objective kit stratification

To provide a rigorous, data-driven, and unsupervised mathematical basis for the performance stratification of the nine commercial kits, we implemented an unsupervised machine learning workflow. A 10-dimensional feature matrix was constructed by integrating five primary analytical performance metrics—including linear recovery points, USP <1132> Guide 1 and Guide 2 effective points, and COA proximity scores—across both model samples (Sample 1 and Sample 2) for all evaluated kits.

Specifically, to eliminate concentration-dependent scale bias and standardize accuracy measurements, the COA proximity score for each kit was quantitatively defined using the following formula:

$$\text{COA proximity score}=10\times \left(1-\frac{\left.{\left|\text{Value}\right.}_{\text{measured}-}{\text{Value}}_{\text{COA}\_\text{target}}\right|}{{\text{Value}}_{\text{COA}\_\text{target}}}\right)$$

Where Value_measured_ represents the residual HCP value quantified by the respective kit under a specific USP guide, and Value COA_target represents the benchmark release specification value provided by the manufacturer (Sample 1 COA median 600 ng/mg, Sample 2 COA 20 ng/mg). For non-responsive dilution regions or data points exceeding the quantitation limits where no reliable value could be determined, the corresponding scores were objectively assigned as 0 to reflect the background baseline. Following Z-score normalization of the entire 10-dimensional matrix to ensure equal weighting across all functional metrics, hierarchical clustering analysis (HCA) utilizing Ward’s linkage method and Euclidean distance was performed. This unsupervised mathematical modeling workflow and the subsequent classification dendrogram were programmatically developed and executed using the Python programming language (Version 3.10), leveraging the scikit-learn library (v1.2) for multivariate data pre-processing, the scipy. cluster.hierarchy module within the SciPy framework (v1.10) for agglomerative clustering computation, and the matplotlib library (v3.7) for high-resolution graphical rendering. The detailed 10-dimensional baseline score matrix utilized for the machine learning input is provided in Supplementary Table S1.

## 4 Results and discussion

### 4.1 Residual HCP results for recombinant protein drug substances 1 and 2 measured by different kits

At present, ELISA is extensively adopted as the industry-standard method for HCP quantification because of its high throughput, operational simplicity, and compatibility with GMP quality systems. In early development, several companies routinely employ commercial HCP ELISA kits; however, during late clinical development or commercial manufacturing, they often switch to process-specific custom HCP ELISA kits. The reasons for developing custom kits include inadequate coverage of the previously utilized commercial kit, lack of specificity for the product process, dilution nonlinearity, or a desire to decrease the risk of missed detection in order to satisfy regulatory expectations. Across industry and regulatory practice, the HCP limit in biologics has traditionally been considered to fall within 1–100 ppm (i.e., 1–100 ng HCP/mg therapeutic protein), a convention that largely arose from the sensitivity limitations of early assays and the analytical capabilities available at that time (Zhu-Shimoni et al., 2014). ELISA yields a single immunologically weighted numerical result whose magnitude is greatly constrained by assay sensitivity, dynamic range, and antibody recognition bias toward specific proteins. Recent USP and EP guidance documents have provided direction and recommendations for monitoring HCP impurities (United States Pharmacopeia, 2017; European Directorate for the Quality of Medicines and HealthCare, 2022). These guidance documents do not specify a single universal numerical threshold because the potential risk associated with HCP exposure depends on several clinical factors, including dose, route of administration, frequency of exposure, indication, patient population, and the specific impurities involved. Dilution linearity is one of the primary indicators for assessing the suitability of an ELISA method for a specific sample because it reflects whether the antibody repertoire recognizes the complex HCP composition in a balanced manner and can show possible antibody insufficiency caused by overabundant individual HCPs. USP <1132> proposes three guides for handling dilution-reported values, especially when nonlinearity is unavoidable, so that the final results can be determined and reported in a scientifically justified manner. Guide 1 recommends averaging all values within 20% of the maximum value, which helps offset random error and reflect overall residual burden. Guide 2 prioritizes removal of lower-dilution samples provided that the CV remains <20%, thereby focusing directly on the elimination of product matrix interference. Guide 3 directly reports the highest value above the quantitation limit and is conservative from a safety-assessment perspective, but it is highly susceptible to error in a single data point. The EP recommends that the final HCP value should be averaged from at least two dilution points that lie within the linear dilution range. If adjacent dilution points differ disproportionately, they cannot both be considered to fall within the linear range. In the present study, nine mainstream commercial kits were systematically assessed to establish a practical kit-screening strategy aligned with international regulatory expectations.

**FIGURE 1:**
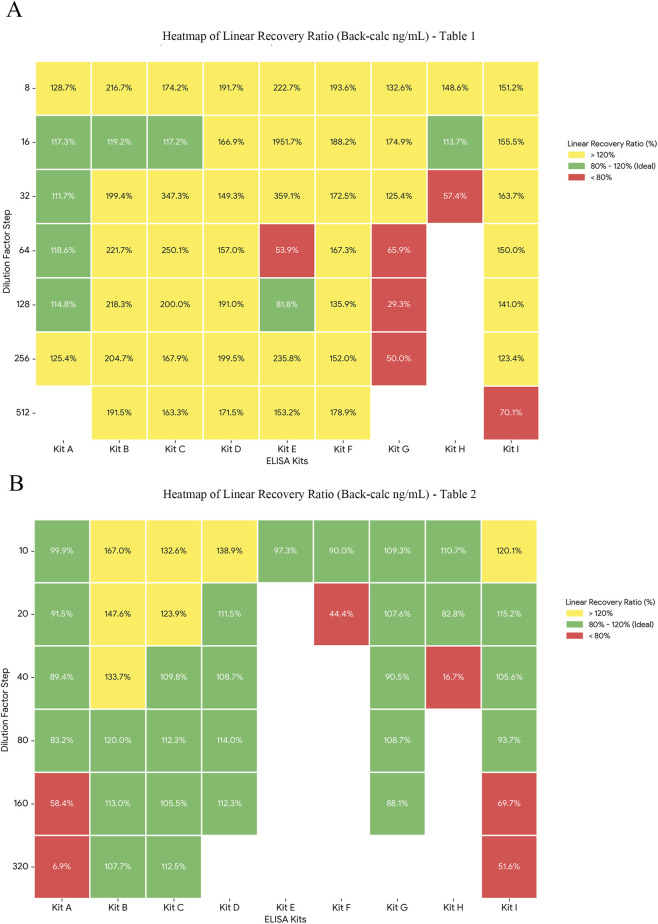
Heatmap analysis of linear back-calculation recovery for nine kits tested with drug substances 1 and 2. **(A)** Heatmap analysis of linear back-calculation recovery for drug substance 1 across nine kits. **(B)** Heatmap analysis of linear back-calculation recovery for drug substance 2 across nine kits.

The nine kits were initially utilized to assess recombinant protein drug substance 1, a CHO cell-derived material at 2.5 mg/mL. The manufacturer employed a self-developed kit for release testing, and the COA specification value was 450–750 ng/mg. HCP levels were measured across a 4-fold to 512-fold dilution range. Based on the adjacent dilution-gradient back-calculation recovery ratio, defined as back-calculation ratio (%) = (HCP concentration_n × dilution factor_n)/(HCP concentration_n-1 × dilution factor_n-1) × 100%, the kits demonstrated marked differences in analytical linearity and stability (Table 1; Figure 1A). Kit A displayed stable back-calculation recovery ratios of 111.7%–118.6% across the 16-fold to 128-fold dilution interval, which fell within the ideal linearity window. In contrast, kits B, C, D, E, F, G, H and I all showed adjacent back-calculation recovery ratios outside the 80%–120% range across the full 4-fold to 512-fold interval. As shown by the large yellow regions in Figure 1A, several ratios substantially exceeded 120%. Notably, kit E reached as high as 1951.7% at the 16-fold dilution. Such anomalous elevation implies severe bias in antibody recognition and indicates a hook effect caused by excessive levels of individual HCP antigens, potentially owing to saturation of antibody binding sites or matrix interference with antibody binding, thereby generating false-negative results (Yanamandra et al., 2025). In other words, highly abundant specific HCPs may competitively hamper formation of the sandwich complex, yielding spuriously low values and indicating uneven affinity distribution or epitope-recognition bias in the antibody repertoire. Kits G and H demonstrated a rapid signal decline at higher dilution factors, suggesting weaker recognition of low-abundance HCPs. According to USP Guides 1 and 2, kits B–F did not meet the calculation requirements, and therefore, the highest value above the quantitation limit was reported (Guide 3). For kits A and I, Guides 1 and 2 each allowed inclusion of two and three dilution points, respectively. Moreover, the residual HCP values acquired with kit A under Guides 1–3 (431.6–656.5 ng/mg) were consistent with the manufacturer COA release range of 450–750 ng/mg, indicating good dilution linearity and good comparability with the original release-testing system.

**TABLE 1:** CHO HCP detection results of recombinant protein drug substance 1 by nine types of detection kits.

Product	Kit A			Kit B			Kit C			Kit D		
R2	1			1			1			1		
Dilution factor	HCP conc. (ng/mL)	HCP ratio (ng/mg)	%Max ratio value	HCP conc. (ng/mL)	HCP ratio (ng/mg)	%Max ratio value	HCP conc. (ng/mL)	HCP ratio (ng/mg)	%Max ratio value	HCP conc. (ng/mL)	HCP ratio (ng/mg)	%Max ratio value
4	178.68	285.9	35%	10.89	17.4	1%	9.60	15.4	1%	18.39	29.4	2%
8	114.97	367.9	45%	11.80	37.8	2%	8.36	26.8	2%	17.63	56.4	4%
16	67.44	431.6	52%	7.03	45.0	3%	4.90	31.4	2%	14.71	94.2	7%
32	37.66	482.0	59%	7.01	89.7	5%	8.51	108.9	7%	10.98	140.6	10%
64	22.33	571.6	69%	7.77	199.0	12%	10.64	272.3	18%	8.62	220.6	15%
128	12.82	656.5	80%	8.48	434.1	25%	10.64	544.7	36%	8.23	421.5	29%
256	8.04	823.5	100%	8.68	889.0	52%	8.93	914.1	61%	8.21	841.0	58%
512	—	—	—	8.31	1702.7	100%	7.29	1493.8	100%	7.04	1442.0	100%
Guide 1	740.0 (n = 2)			—			—			—		
Guide 2	683.9 (n = 3)			—			—			—		
Guide 3	823.5 (n = 1)			1702.7 (n = 1)			1493.8 (n = 1)			1442.0 (n = 1)		

Kit E			Kit F			Kit G			Kit H			Kit I		
1			1			1			1			1		
HCP conc. (ng/mL)	HCP ratio (ng/mg)	%Max ratio value	HCP conc. (ng/mL)	HCP ratio (ng/mg)	%Max ratio value	HCP conc. (ng/mL)	HCP ratio (ng/mg)	%Max ratio value	HCP conc. (ng/mL)	HCP ratio (ng/mg)	%Max ratio value	HCP conc. (ng/mL)	HCP ratio (ng/mg)	% Max ratio value
7.48	12.0	0.4%	2.80	4.5	3%	6.85	11.0	34%	8.17	13.1	59%	8.73	14.0	10%
8.33	26.7	0.9%	2.71	8.7	5%	4.54	14.5	46%	6.07	19.4	88%	6.60	21.1	15%
81.29	520.2	17%	2.55	16.3	9%	3.97	25.4	80%	3.45	22.1	100%	5.13	32.9	23%
145.96	1868.3	63%	2.20	28.1	16%	2.49	31.8	100%	0.99	12.6	57%	4.20	53.8	38%
39.35	1007.3	34%	1.84	47.0	27%	0.82	20.9	66%	—	—	—	3.15	80.6	58%
16.10	824.1	28%	1.25	63.8	37%	0.12	6.1	19%	—	—	—	2.22	113.7	81%
18.98	1943.4	65%	0.95	97.7	56%	0.03	3.1	10%	—	—	—	1.37	139.9	100%
14.54	2977.8	100%	0.85	173.5	100%	—	—	—	—	—	—	0.48	98.5	70%
—			—			28.6 (n = 2)			20.7 (n = 2)			126.8 (n = 2)		
—			—			28.6 (n = 2)			—			117.4 (n = 3)		
2977.8 (n = 1)			173.5 (n = 1)			31.83 (n = 1)			22.1 (n = 1)			139.9 (n = 1)		

Guide 1 states that all values within 20% of the maximum value are averaged. Guide 2 states that values are averaged as long as the CV of the values is < 20%, removing less diluted samples first. Guide 3 states to report the highest value measured above the QL but the suitability of this approach should be very well demonstrated by method validation. Nonlinear data; the Guide value is provided for reference only.

In order to further confirm kit suitability for samples with varying HCP burdens, recombinant protein drug substance 2 (30 mg/mL) was tested over a 5-fold to 320-fold dilution range (Table 2; Figure 1B). This product is currently released utilizing commercial kit C, with a mean COA value of approximately 20 ng/mg. Based on adjacent dilution-gradient back-calculation recovery, kits A, B, C, D, G, and I each provided at least three adjacent dilution points within the 80%–120% acceptance window across the 5-fold to 320-fold range, revealing good adaptability to high-concentration samples. By contrast, kits E, F, and H exhibited signals below the limit of detection at low dilution factors, together with marked fluctuations in back-calculation recovery. For example, kit H decreased sharply to 16.7% at the 40-fold dilution, indicating inadequate sensitivity for residual-HCP testing in highly purified samples. According to USP Guides 1 and 2, kits A, C, G, and I covered relatively more dilution points (≥4 points). Relative to the manufacturer COA value, the value obtained with kit G was biased low, whereas Guides 1–3 values obtained with kits A and I were close to the release result obtained with kit C, the kit used for COA generation, at approximately 20 ng/mg.

**TABLE 2:** CHO HCP detection results of recombinant protein drug substance 2 by nine types of detection kits.

Product	Kit A			Kit B			Kit C			Kit D		
R2	1			1			1			1		
Dilution factor	HCP conc. (ng/mL)	HCP ratio (ng/mg)	%Max ratio value	HCP conc. (ng/mL)	HCP ratio (ng/mg)	%Max ratio value	HCP conc. (ng/mL)	HCP ratio (ng/mg)	%Max ratio value	HCP conc. (ng/mL)	HCP ratio (ng/mg)	%Max ratio value
5	116.97	19.5	100%	27.9	4.7	21%	71.8	12.0	42%	47.5	7.9	46%
10	58.43	19.5	100%	23.3	7.8	35%	47.6	15.9	56%	33.0	11.0	64%
20	26.72	17.8	91%	17.2	11.4	51%	29.5	19.7	70%	18.4	12.3	72%
40	11.95	15.9	82%	11.5	15.3	69%	16.2	21.7	77%	10.0	13.3	78%
80	4.97	13.2	68%	6.9	18.4	82%	9.1	24.2	85%	5.7	15.3	89%
160	1.45	7.7	39%	3.9	20.8	94%	4.8	25.8	91%	3.2	17.1	100%
320	0.05	0.52	3%	2.1	22.3	100%	2.7	28.3	100%	—	—	—
Guide 1	18.2 (n = 4)			20.5 (n = 3)			25.0 (n = 4)			15.2 (n = 3)		
Guide 2	17.2 (n = 5)			20.5 (n = 3)			22.6 (n = 6)			13.8 (n = 5)		
Guide 3	19.5			22.3			28.3			17.1		

Kit E			Kit F			Kit G			Kit H			Kit I		
1			1			1			1			1		
HCP conc. (ng/mL)	HCP ratio (ng/mg)	%Max ratio value	HCP conc. (ng/mL)	HCP ratio (ng/mg)	%Max ratio value	HCP conc. (ng/mL)	HCP ratio (ng/mg)	%Max ratio value	HCP conc. (ng/mL)	HCP ratio (ng/mg)	%Max ratio value	HCP conc. (ng/mL)	HCP ratio (ng/mg)	%Max ratio value
3.7	0.6	100%	4.0	0.7	100%	40.8	6.8	85%	26.2	4.4	90%	104.1	17.4	68%
1.8	0.6	95%	1.8	0.6	89%	22.3	7.4	93%	14.5	4.8	100%	62.5	20.8	82%
< LOQ	< LOQ	—	0.4	0.3	38%	12.0	8.0	100%	6.0	4.0	83%	36.0	24.0	95%
< LOQ	< LOQ	—	< LOQ	< LOQ	< LOQ	5.43	7.23	90%	0.5	0.7	15%	19.0	25.3	100%
< LOQ	< LOQ	—	< LOQ	< LOQ	< LOQ	2.95	7.87	98%	< LOQ	< LOQ	< LOQ	8.9	23.6	93%
< LOQ	< LOQ	—	< LOQ	< LOQ	< LOQ	1.30	6.94	87%	< LOQ	< LOQ	< LOQ	3.1	16.8	66%
< LOQ	< LOQ	—	< LOQ	< LOQ	< LOQ	< LOQ	< LOQ	< LOQ	< LOQ	< LOQ	< LOQ	0.8	8.1	32%
0.6 (n = 2)			0.6 (n = 2)			7.4 (n = 6)			4.4 (n = 3)			23.4 (n = 4)		
0.6 (n = 2)			0.6 (n = 2)			7.4 (n = 6)			4.4 (n = 3)			21.3 (n = 6)		
0.6			0.7			8.0			4.8			25.3		

Guide 1 states that all values within 20% of the maximum value are averaged. Guide 2 states that values are averaged as long as the CV of the values is < 20%, removing less diluted samples first. Guide 3 states to report the highest value measured above the QL but the suitability of this approach should be very well demonstrated by method validation. Nonlinear data; the Guide value is provided for reference only.

**FIGURE 2:**
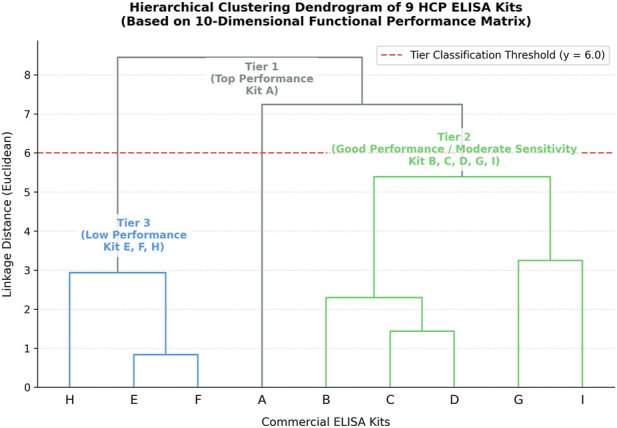
Hierarchical clustering dendrogram of nine commercial CHO HCP ELISA kits based on a 10-dimensional functional performance matrix. The red dashed line denotes the tier classification threshold at a linkage distance of 6.0.

Using the sequential criteria of adjacent dilution-gradient back-calculation recovery (80%–120%), the number of dilution points covered under USP <1132>, and agreement with the manufacturer COA value, supplemented by the objective mathematical distances from our unsupervised hierarchical clustering analysis (HCA, cut-off at y = 6.0) (Figure 2), the nine kits demonstrated obvious performance stratification across the two DS samples, representing high and low residual HCP burdens, respectively. The first-tier kit (Kit A) showed the broadest and most stable adjacent dilution-gradient back-calculation recovery window. It successfully accommodated both samples despite their distinct origins and markedly distinctive HCP levels, demonstrating reliable matrix tolerance for the high-burden sample and good analytical sensitivity for the highly purified sample. The hierarchical clustering analysis (HCA) clearly separated Kit A into a unique branch, mathematically confirming its superior and consistent performance across samples with varying HCP burdens. Second-tier kits (kits B, C, D, G and I) demonstrated a certain linear range and moderate sensitivity, but their quantitative results were more sensitive to the choice of dilution factor. As shown in Table 1, many nonlinear regions were associated with back-calculation recovery ratios >120%. The algorithm grouped these five kits into a second tier because they share a common performance limitation: while they perform adequately on low-burden samples, they remain highly susceptible to severe matrix interference or hook effects when testing samples with high HCP impurities. Although MRD determination is a standard procedure for all HCP assays, rigorous validation of an appropriate MRD is particularly critical for these kits to mitigate matrix interference prior to routine use. Third-tier kits (kits E, F and H) showed pronounced nonlinear response and/or insufficient sensitivity, signifying intrinsic limitations in antibody-recognition characteristics or assay kinetics. The clustering model grouped Kit H with the lower-performing Kits E and F rather than the middle-tier kits, demonstrating that Kit H consistently exhibited poor functional performance across all evaluated metrics. Prior studies have reported that both the specificity and breadth of the antibody repertoire are critical for accurate HCP detection (Baldus et al., 2018), and the poor performance observed here may also be related to the composition of the antibody repertoire or mismatch between the standard and the actual HCP profile of the sample.

Notably, the numerical discrepancies seen among the nine kits for the same sample were further quantified utilizing the percent coefficient of variation (CV%). At the 64-fold dilution of drug substance 1, the measured residual HCP levels ranged from 20.9 ng/mg to 1007.3 ng/mg, corresponding to an approximately 48-fold spread, which underscores the substantial method-dependent bias among kits. It should be noted that some low values, such as the 20.9 ng/mg obtained with kit G, were close to the LOQ and may therefore have considerable measurement uncertainty. At the 40-fold dilution of drug substance 2, the CV% was 58.6%, and the extreme-value spread was approximately 36-fold. The root cause of these differences among commercial kits lies in the fact that polyclonal antibody generation depends on animal immune responses, and different animals vary in their immunogenic responsiveness to different HCPs, resulting in unequal abundances of antibodies against specific HCPs within the repertoire. Thus, the pronounced method-dependent bias observed here reflects antibody-repertoire heterogeneity arising from differences in immunogen source, host animal species, and purification process among commercial kits. This major inconsistency in numerical results also indicates that switching HCP testing kits during the product life cycle should be approached with caution. Any replacement lot or new kit should outperform the original kit in key characteristics, such as coverage, sensitivity, and resistance to matrix interference, so that continued accurate assessment of process clearance capability can be ensured in compliance with process-transfer and regulatory expectations. Our results also strongly support the importance of establishing national HCP reference standards or universal process quality-control materials (Graham et al., 2026). A unified reference scale would not only calibrate the recognition intensity across various antibody systems and decrease method-dependent bias, but also provide an authoritative bridging basis when companies change quality-control kits. Additionally, unified control materials could enforce a minimum recognition threshold for key high-risk proteins in commercial kits, thereby reducing immunogenicity risk at the source and enhancing the scientific rigor and traceability of residual HCP monitoring in biologics (Coye et al., 2025).

### 4.2 Analytical method validation of kit A

The U.S. Food and Drug Administration (FDA) follows the requirements for biotechnology/biological product specifications described in ICH Q6B, which include HCP analysis. The FDA urges a risk-based approach to HCP analysis, meaning that the depth of analysis and the required rigor may depend on product characteristics, intended use, and potential impact on patient safety. HCP methods must be authenticated, and the validation should show specificity, sensitivity, precision, and accuracy. Given the favorable performance of kit A under the screening conditions, this kit was chosen for subsequent comprehensive analytical validation.

#### 4.2.1 Specificity

No signal was revealed in the sample diluent, and the spike recovery in diluent was 100%, showing suitable method specificity and no evident matrix interference.

#### 4.2.2 Linearity

**FIGURE 3:**
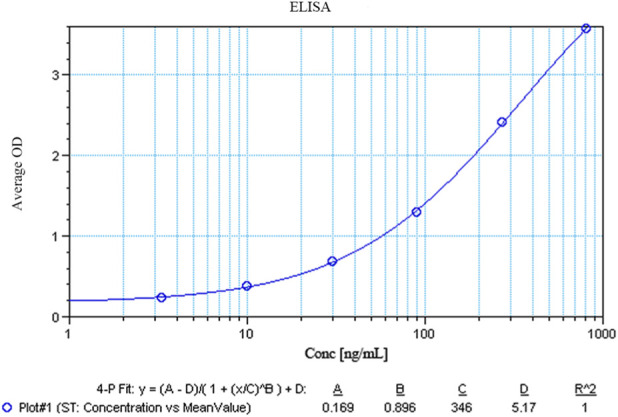
Fitted standard curve.

The standard curve exhibited a four-parameter fit with R^2^ ≥ 0.990 over the concentration range of 3.3–810 ng/mL (Figure 3), implying a satisfactory dose-response relationship within this range and suitability for quantitative analysis.

#### 4.2.3 Precision

Within-run precision: Drug substance 1 diluted 128-fold was measured six times in parallel, yielding a CV of 6.7%.

Between-run precision: A 16-fold diluted sample was measured on three different analytical days, yielding a CV of 8.3%. Both values were below 20%, indicating suitable repeatability and intermediate precision (Table 3).

**TABLE 3:** Precision validation results.

Sample/Dilution	Within-run precision (n = 6)			Sample/Dilution	Between-run precision (n = 3)		
	Mean (ng/mL)	SD	CV (%)		Mean (ng/mL)	SD	CV (%)
128	15.85	1.056	6.7	16	66.34	5.481	8.3

Abbreviations: SD, standard deviation; CV, coefficient of variation.

#### 4.2.4 Accuracy

In order to assess the method accuracy, two complementary authentication approaches were employed. First, standard-spike recovery was evaluated at three concentration levels, 3.3, 90, and 810 ng/mL, representing low, medium, and high ranges, respectively. The measured recoveries were 84%, 94%, and 103% (Table 4) for 3.3, 90, and 810 ng/mL, respectively, and the corresponding CVs were all well controlled. Second, spike-recovery testing was conducted for recombinant protein drug substance 1 across a broad dilution range from 16-fold to 256-fold. The results demonstrated that spike recoveries remained stably within 80%–120% throughout this dilution interval. These data indicate that the method had excellent accuracy within the target linear range and was not subject to substantial interference from the product matrix.

**TABLE 4:** Accuracy validation results.

Standard concentration (ng/mL)	Measured value (ng/mL)	Recovery (%)	CV (%)
810	836.2	103	0.1
90	84.8	94	0.2
3.3	2.8	84	8.7

Abbreviations: SD, standard deviation; CV, coefficient of variation.

#### 4.2.5 Spike recovery under dilution linearity and reported values

Analysis was conducted according to the three averaging guides recommended in USP <1132> (Table 5):

**TABLE 5:** Spike-recovery results.

Batch	Dilution factor	Measured value (ng/mL)	Spike recovery (%)	Residual HCP (ng/mg)	Mean guide 1 (ng/mg)	Mean guide 2 (ng/mg)	Mean guide 3 (ng/mg)
Batch 1	16	81.58	102	522	640 (n = 3)	582 (n = 5)	688
	32	36.559	104	468			
	64	21.356	108	547			
	128	13.358	97	684			
	256	6.723	94	688			
Batch 2	16	87.399	118	559	706 (n = 3)	638 (n = 5)	766
	32	40.141	116	514			
	64	23.859	112	611			
	128	14.501	112	742			
	256	7.479	111	766			
Batch 3	16	71.20	117	456	689 (n = 4)	643 (n = 5)	773
	32	45.20	100	579			
	64	26.27	104	672			
	128	14.32	99	733			
	256	7.545	105	773			
Diluent	—	—	—	—	—	—	—

Guide 1 (average of values within 20% of the maximum): Kit A demonstrated suitable concentration dependence over the 16-fold to 256-fold dilution range, corresponding to residual HCP values of 431.6–823.5 ng/mg. The Guide 1 mean values for the three batches were 640, 706, and 689 ng/mg.

Guide 2 (CV < 20%, with removal of lower-dilution samples first): the results for batches 1–3 were 582 ng/mg (n = 5), 638 ng/mg (n = 5), and 643 ng/mg (n = 5), respectively.

Guide 3 (report the highest value above the quantitation limit): the back-calculated values at the 256-fold dilution were reported directly as 688, 766, and 773 ng/mg.

The high consistency of the results acquired with kit A under all three principles further confirms the robustness of the method. For samples displaying obvious matrix interference or nonlinear response at low dilution factors, the MRD should be systematically established. The MRD is determined by spiking HCP standard into a dilution series of the sample—for example, 2-fold serial dilutions starting from the neat sample, then measuring spike recovery at each dilution. The smallest dilution factor at which spike recovery remains stable within 70%–130%, or 50%–150% when close to the quantitation limit, is defined as the MRD. In subsequent testing, all samples should be diluted to at least the MRD to ensure sufficient elimination of matrix effects and accurate measurement. For kit A, spike recoveries for drug substance 1 remained stable within 80%–120% across the 16-fold to 256-fold dilution interval (Table 5), signifying that the MRD can be set at 16-fold. For kits that show severe deviation in recovery or obvious nonlinearity at low dilution factors, a higher MRD or replacement with a more suitable kit should be considered. Additionally, whenever the manufacturing process is changed or a new kit lot is introduced, the MRD should be revalidated to ensure continued method suitability.

### 4.3 IMBS-MS/MS-based evaluation of antibody coverage and identification of high-risk proteins

**FIGURE 4:**
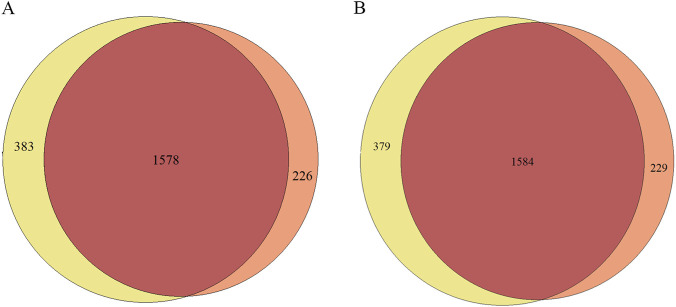
Venn diagrams of antibody coverage for kits A and I. **(A)** Venn diagram of antibody coverage for kit A. **(B)** Venn diagram of antibody coverage for kit I. The left yellow area denotes proteins detected exclusively in the unenriched HCP sample. The middle red area represented proteins common to both samples. The right orange area comprises proteins detected exclusively in the enriched HCP sample.

**FIGURE 5:**
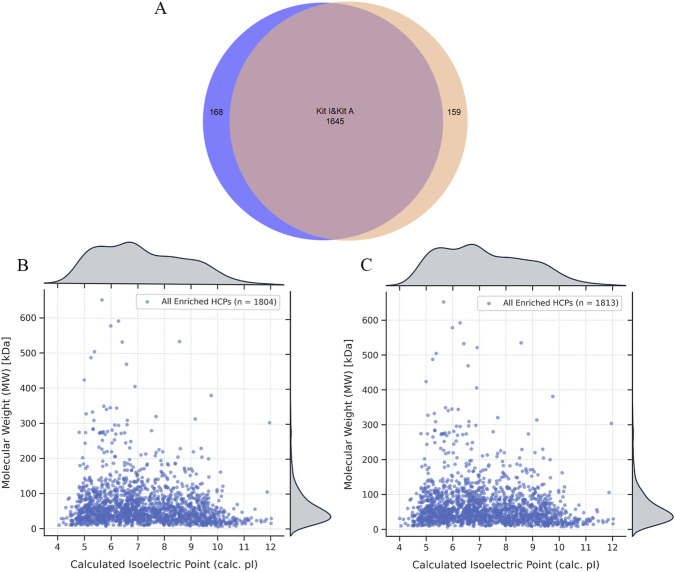
Comparative host–protein recognition by kits A and I. **(A)** Venn diagram of total enriched HCPs detected by kits A and I. **(B)** Physicochemical-property profiling (Molecular Weight vs. Calculated Isoelectric Point) of total enriched proteins for Kit A (n = 1804) **(C)** Physicochemical-property profiling (Molecular Weight vs. Calculated Isoelectric Point) of total enriched proteins for Kit I (n = 1813).

ELISA detects HCPs by utilizing polyclonal antibodies raised in animals immunized with HCP mixtures derived from null cell lines (Jin et al., 2010). Prior studies have demonstrated that the HCP composition of null CHO cell lines is similar to that of cell lines employed for the production of biotherapeutics (Obrstar, et al., 2018). This similarity suggests that antibodies developed using null-cell-line materials are likely to represent the HCP pool present in recombinant production cell lines reasonably well (Tait et al., 2012). Although two-dimensional Western blotting has long been regarded as the industry-standard method for assessment of HCP antibody coverage, it has intrinsic limitations, particularly with respect to epitope recognition after protein denaturation/reduction and detection of low-abundance proteins. In the current study, IMBS-MS/MS was used as an orthogonal evaluation tool. This strategy compensates for the limited separation capability of traditional two-dimensional electrophoresis and enables deep proteomic identification of high-risk proteins, thereby offering more precise evidence regarding the recognition depth of kit antibody repertoires. In order to assess the mechanistic basis underlying the functional differences observed among kits, the best-performing kit A, representing a first-tier kit, and kit I, which performed relatively well among the second-tier kits in the screening of both drug substances 1 and 2, were chosen for empirical comparison of antibody coverage. Capture and identification of total HCPs from CHO cells demonstrated that kits A and I achieved protein-level coverage of 82.5% (calculated as (226 + 1578)/(226 + 1578 + 383) = 82.5%) and 82.7% (calculated as (229 + 1584)/(229 + 1584 + 379) = 82.7%), respectively (Figures 4A,B). Both values exceeded 80%, implying broad HCP-recognition capability consistent with regulatory expectations for broad immunoassay coverage. As shown in Figure 5A, both kits A and I displayed highly overlapping recognition spectra for CHO HCP identification, with common proteins accounting for 83.4% of the total identified proteins, indicating strong concordance in broad recognition capability between the two antibody repertoires. However, despite their similar breadth of recognition, the dilution-linearity and accuracy experiments described above (Sections 4.1, 4.2) showed that kit A maintained back-calculation recovery ratios stably within 80%–120% across various dilution factors, whereas kit I demonstrated more obvious matrix interference and nonlinear response when handling highly concentrated samples. These results indicate that breadth of recognition alone is not a sufficient condition for robust ELISA performance. Functional performance also depends on antibody affinity toward specific HCPs, epitope-recognition capability, and the compositional balance of antibody concentrations within the repertoire. Further analysis of physicochemical-property distributions revealed that the core protein populations enriched by the two kits were highly similar in central tendency (Figures 5B,C; Supplementary Figure S1). In terms of molecular weight (MW), the target proteins exhibited pronounced clustering in the low-to-medium MW range of 20–80 kDa, with a median of approximately 50 kDa. In terms of isoelectric point (pI), a typical bimodal distribution was observed, with major enrichment in the mildly acidic to neutral range (pI 5.0–7.5) and a secondary cluster in the alkaline region (pI 8.5–10.0). Despite this high degree of macroscopic similarity in enrichment behavior, weak preferential differences were observed for a small subset of kit-specific proteins: kit A displayed slightly denser representation in the very-low-MW region (<30 kDa), whereas kit I showed relatively more dispersion in the medium-to-high-MW range (80–150 kDa). To further interrogate this difference, the captured-protein lists were compared with a published panel of 38 high-risk CHO HCPs (Xu et al., 2025). Both kits A and I successfully captured phospholipase B-like protein 2 (PLBL2), a highly immunogenic and difficult-to-clear HCP, as well as clusterin (CLU), which is prone to co-purifying with recombinant proteins through noncovalent interactions (Table 6). In addition, Cathepsin D is particularly remarkable because its physicochemical properties resemble those of the target product, making it difficult to remove completely during downstream purification, while its proteolytic activity may directly cause product fragmentation. Under the latest quality-risk frameworks, identification of such high-risk proteins represents a crucial starting point for clinical safety evaluation (Coye et al., 2025). For example, although PLBL2 did not cause severe adverse events in clinical studies of lebrikizumab, the very high rate of antibody generation, 76%–90%, warns that even extremely low residual levels of a highly immunogenic HCP may materially affect safety assessments (Fischer et al., 2017), as *in vitro* data show PLBL2 can slightly increase dendritic cell maturation markers, suggesting an adjuvant potential that warrants continued monitoring (Panikulam et al., 2025). Therefore, selecting kits capable of stably detecting such high-risk proteins is crucial for decreasing unknown risks in downstream clinical development.

**TABLE 6:** Summary of high-risk proteins identified by kits A and I.

Accession	Protein name	MW (kDa)	Detection	Adverse effects
G3HRK9	Matrix metallopeptidase 19	58.94	Kit a+ kit I	Degradation of the drug
G3HKV9	Group XV phospholipase A2	47.23	Kit a+ kit I	Degradation of polysorbates^1^
2G3HN89	Palmitoyl-protein thioesterase 1	34.56	Kit a+ kit I	Degradation of polysorbates
G3HQY6	Lipase	45.63	Kit a+ kit I	Degradation of polysorbates^1^
G3H0L9	Cathepsin B	37.50	Kit a+ kit I	Fragmentation of the drug^2^
G3I4W7	Cathepsin D	44.11	Kit a+ kit I	Fragmentation of the drug^2^
G3INC5	Cathepsin L1	37.24	Kit a+ kit I	Fragmentation of the drug^2^
Q9EPP7	Cathepsin Z	34.02	Kit a+ kit I	Fragmentation of the drug^2^
G3GTT2	C-C motif chemokine	15.85	Kit a+ kit I	Immunogenic response^3^
G3H7R4	Peroxiredoxin 3	28.12	Kit A	Immunogenic response^3^
G3HC31	Protein S100	10.05	Kit a+ kit I	Immunogenic response^3^
G3HNJ3	Clusterin	51.75	Kit a+ kit I	Immunogenic response^3^
G3I6T1	Phospholipase B-like 2	65.54	Kit a+ kit I	Immunogenic response^3^ ^,^ ^4^

1 Weiss et al. (2024).

2 Dovgan et al. (2021).

3 Panikulam et al. (2025).

4 Fischer et al. (2017).

Further comparative analysis revealed that, among the identified high-risk proteins (Table 6), kit A recognized and covered 13 key high-risk proteins, demonstrating broader coverage and stronger component-capture capability. Notably, all high-risk proteins detected by kit I were sequence-wise a subset of those detected by kit A, with only a slightly smaller number, 12 proteins, likely attributable to sensitivity differences. However, the distinct differences between the two residual-protein profiles indicate that the monitoring capability of an HCP kit for critical risk proteins depends not only on antigen coverage, but also on antibody affinity for low-abundance proteins in complex matrices, recognition patterns for specific epitopes, and the compositional balance of the polyclonal antibody repertoire (Seisenberger et al., 2021; Seisenberger et al., 2023). It is also significant to note that the coverage samples utilized in this study originated from a null cell line, whereas the HCP profile in actual manufacturing may be more complex. Previous studies have demonstrated that, as compared with null cell lines, monoclonal antibody-producing cell lines can display substantial changes in harvested HCP profiles owing to cellular stress and metabolic alterations, including increased levels of intracellular proteins, such as lactate dehydrogenase and protein disulfide isomerase (PDI), suggesting that HCPs in harvest material may arise more from cell breakage and lysis than from active secretion (Quarmby et al., 2012). In production cell lines, proteins involved in folding, such as PDI, may be induced, whereas this is not seen in null cell lines. Thus, the product molecule itself may play an important role in influencing the HCP profile. Induced proteins may promote correct product folding through interaction with the product protein. Such HCP-product interactions may render certain impurities difficult to detect by conventional methods, giving rise to the so-called “hitchhiker proteins” (Bee et al., 2015).

In summary, the orthogonal-validation model combining gold-standard ELISA with proteomic IAC-MS/MS confirms the broadness of kit antibody repertoires and identifies the necessary yet insufficient relationship between high coverage and functional performance. Kits that combine high antibody coverage with favorable functional performance, particularly with respect to linearity and accuracy, are better suited as preferred tools for high-standard residual-HCP testing in biologics. This finding is highly consistent with the view proposed by Henry et al. (2017), namely, that the coverage assessment of ELISA kits should focus on high-risk HCPs that remain after downstream processing, may co-purify with the product, and can affect product quality, rather than simply maximizing total HCP coverage. Our findings indicate that kit selection and validation should adopt a combined strategy of functional testing plus coverage analysis, with particular emphasis on recognition capability for process-specific HCPs, so that the HCP method accurately reflects process-clearance performance and product risk. In the future, integrating the high-throughput monitoring capability of ELISA with the comprehensive analytical power of proteomics to develop monitoring strategies for key risk HCPs is likely to become a vital direction for lean manufacturing and precise quality control of biopharmaceuticals.

Moving beyond the final purified Drug Substance (DS), the implications of our findings extend significantly to the testing and monitoring of downstream in-process intermediates. In contrast to the purified DS, in-process intermediates harbor orders-of-magnitude higher HCP concentrations and immense molecular heterogeneity. First, the high abundance of background proteins in early purification harvests substantially heightens the risk of multi-antigen saturation and hook effects, reinforcing the critical need to rigorously evaluate dilution linearity across a wider dynamic range during in-process control (IPC). Second, because specific high-risk HCPs (e.g., lipases or hitchhiker proteins) coexist with thousands of transient impurities in intermediates, an ELISA kit with insufficient antibody coverage or binding affinity might suffer from competitive inhibition, masking the true clearance kinetics of critical risk proteins (Ducry et al., 2020). Therefore, understanding the distinct recognition profiles and methodological limitations of different commercial kits is paramount for process engineers to select orthogonal, assay-specific monitoring tools across different chromatography stages, ultimately ensuring a well-characterized and robust purification process (Falkenberg et al., 2019).

Regarding the generalizability of our orthogonal evaluation strategy, the framework itself—combining adjacent dilution-gradient back-calculation recovery with proteomic IMBS-MS/MS characterization—is inherently universal and directly applicable to other modalities, including monoclonal antibodies (mAbs) (Gunawan et al., 2018). However, the specific ranking and performance profiles of commercial kits must be implemented on a case-by-case basis for different bioprocesses. mAbs often exhibit distinct host cell protein (HCP) profiles due to product-specific interactions (such as the ‘hitchhiker effect’ where certain HCPs non-covalently bind to the mAb matrix) and platform-specific purification processes (Van de Velde et al., 2020). Therefore, while researchers can confidently adopt our integrated workflow to screen kits for mAb programs, they should not directly transplant the numerical performance rankings of specific kits from this study without process-specific empirical validation (Zarei et al., 2024).

## 5 Conclusion

Based on the findings in this study, the following practical recommendations are proposed for the selection and application of HCP kits during biologics development: 1) The robustness of dilution linearity should be used as an auxiliary criterion for kit screening. During the early screening stage, at least two representative process-step samples should be selected for dilution-gradient testing, and priority should be given to kits whose back-calculation recovery ratios remain stable within 80%–120% over a broad dilution range. 2) Matrix interference at low dilution factors should be monitored. If obvious nonlinearity or abnormal recovery is observed at low dilutions, the MRD should be established according to USP <1132> to eliminate matrix effects. 3) Coverage and functional indicators should be integrated. High coverage is not equivalent to high performance. On the basis of adequate coverage, the linear performance of the kit in the actual processing of samples should be prioritized. 4) Method stability should be dynamically monitored. Because HCP composition is process-related, whenever major upstream variations (e.g., cell culture stress, viability fluctuations) (Sahoo et al., 2024) or downstream purification modifications (e.g., chromatography chemistry alterations or host-protein hitchhiking) occur (Bhoyar et al., 2025), the kit-screening strategy established in this study should be utilized to re-evaluate the suitability of the available kit.

This study established a novel quality-evaluation strategy for HCP detection kits utilized in biologics by integrating adjacent dilution-gradient back-calculation recovery with orthogonal immunocapture-mass spectrometric analysis. The framework provides a practical technical pathway for biopharmaceutical companies to make scientifically sound and efficient choices among numerous commercial kits, and it also offers data support for the future development of HCP-related guidance in the Chinese Pharmacopoeia. Going forward, combining the high-throughput monitoring capability of ELISA with the in-depth analytical advantages of proteomics to develop monitoring strategies for key risk HCPs is expected to become a significant technical approach for the precise life-cycle quality control of biologics.

## Data Availability

The raw data supporting the conclusions of this article will be made available by the authors, without undue reservation.
