# Supplementary material for: A quality evaluation strategy for residual host cell proteins based on orthogonal analysis
Source: Front Bioeng Biotechnol. 2026 Jul 14;14:1886078. doi: 10.3389/fbioe.2026.1886078 (PMC13407541; doi:10.3389/fbioe.2026.1886078)
Supplement: Supplementary file 1 [file Supplementaryfile1.docx]

#### Supplementary Material: Unsupervised Machine Learning Dataset for Kit Performance Stratification

##### Section S1. Description of the 10-Dimensional Multivariate Matrix

To completely eliminate human bias and mathematically validate the performance stratification of the nine commercial CHO HCP ELISA kits, an unsupervised Hierarchical Clustering Analysis (HCA) was performed (as cross-referenced in Section 4.1 and Section 3.4 of the main text). The machine learning algorithm was fed with a comprehensive 10-dimensional multivariate matrix constructed from five key functional performance parameters evaluated across both distinct model samples (Sample 1 with a high HCP burden and Sample 2 with a low HCP burden). The definitions of the five core parameters used to establish the feature vectors for each kit are as follows:

**Linear Recovery Points:** The total number of adjacent dilution steps where the back-calculation recovery ratio was strictly maintained within the ideal acceptance window of 80%–120%. This metric reflects a kit’s robust analytical linearity and resistance to matrix interference.

**USP Guide 1 Points:** The number of valid dilution data points that met the calculation requirements under USP <1132> Guide 1 (averaging all values within 20% of the maximum reported concentration).

**USP Guide 2 Points:** The number of valid dilution data points covered under USP <1132> Guide 2 (averaging values with an intermediate precision CV < 20%, sequentially removing lower-dilution data points to eliminate product matrix interference).

**COA Proximity Score (Guide 1):** A normalized accuracy score scaling from 0 to 10, quantifying how closely the final reported HCP value calculated via USP Guide 1 matched the manufacturer's official Certificate of Analysis (COA) release benchmark. It was calculated using the formula:


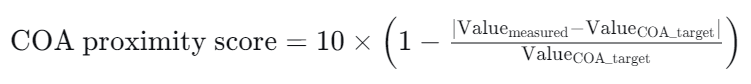


**COA Proximity Score (Guide 2):** A normalized accuracy score scaling from 0 to 10, quantifying the proximity of the reported HCP value calculated via USP Guide 2 to the manufacturer's COA benchmark using the identical mathematical proximity formulation.

*Note on Data Pre-processing:* For unresponsive dilution windows, highly non-linear regions, or extreme data points exceeding the lower/upper limits of quantitation where USP guidelines could not be computationally fulfilled (originally denoted as "/" in raw datasets), the corresponding metrics were objectively assigned a baseline value of 0. Before applying the Ward’s linkage clustering algorithm, the entire 10-dimensional matrix was subjected to *Z*-score standardization to ensure that all functional parameters contributed with equal mathematical weight.

##### Supplementary Table S1. Normalized Functional Performance Feature Matrix of 9 Commercial Kits Used for Hierarchical Clustering Analysis

| **Kit Code** | **S1_Linear** | **S1_Guide1** | **S1_Guide2** | **S1_COA** | **S1_COA** | **S2_Linear** | **S2_Guide1** | **S2_Guide2** | **S2_COA** | **S2_COA** |
| --- | --- | --- | --- | --- | --- | --- | --- | --- | --- | --- |
| **Kit A** | 4.0 | 2.0 | 3.0 | 7.67 | 8.60 | 4.0 | 4.0 | 5.0 | 9.10 | 8.60 |
| **Kit B** | 1.0 | 0.0 | 0.0 | 0.00 | 0.00 | 3.0 | 3.0 | 3.0 | 9.75 | 9.75 |
| **Kit C** | 1.0 | 0.0 | 0.0 | 0.00 | 0.00 | 4.0 | 4.0 | 6.0 | 7.50 | 8.70 |
| **Kit D** | 0.0 | 0.0 | 0.0 | 0.00 | 0.00 | 4.0 | 3.0 | 5.0 | 7.60 | 6.90 |
| **Kit E** | 1.0 | 0.0 | 0.0 | 0.00 | 0.00 | 1.0 | 2.0 | 2.0 | 0.30 | 0.30 |
| **Kit F** | 0.0 | 0.0 | 0.0 | 0.00 | 0.00 | 1.0 | 2.0 | 2.0 | 0.30 | 0.30 |
| **Kit G** | 0.0 | 2.0 | 2.0 | 0.48 | 0.48 | 5.0 | 6.0 | 6.0 | 3.70 | 3.70 |
| **Kit H** | 1.0 | 2.0 | 0.0 | 0.35 | 0.00 | 2.0 | 3.0 | 3.0 | 2.20 | 2.20 |
| **Kit I** | 0.0 | 2.0 | 3.0 | 2.11 | 1.96 | 3.0 | 4.0 | 6.0 | 8.30 | 9.35 |

*Abbreviations: S1 = Sample 1 (High HCP Burden); S2 = Sample 2 (Low HCP Burden); Linear_Rec = Adjacent dilution-gradient back-calculation recovery points within 80%–120%; Pts = Covered dilution points under specified USP guides; COA_Prox = Calculated proximity score to the manufacturer’s certificate-of-analysis release value.*

*
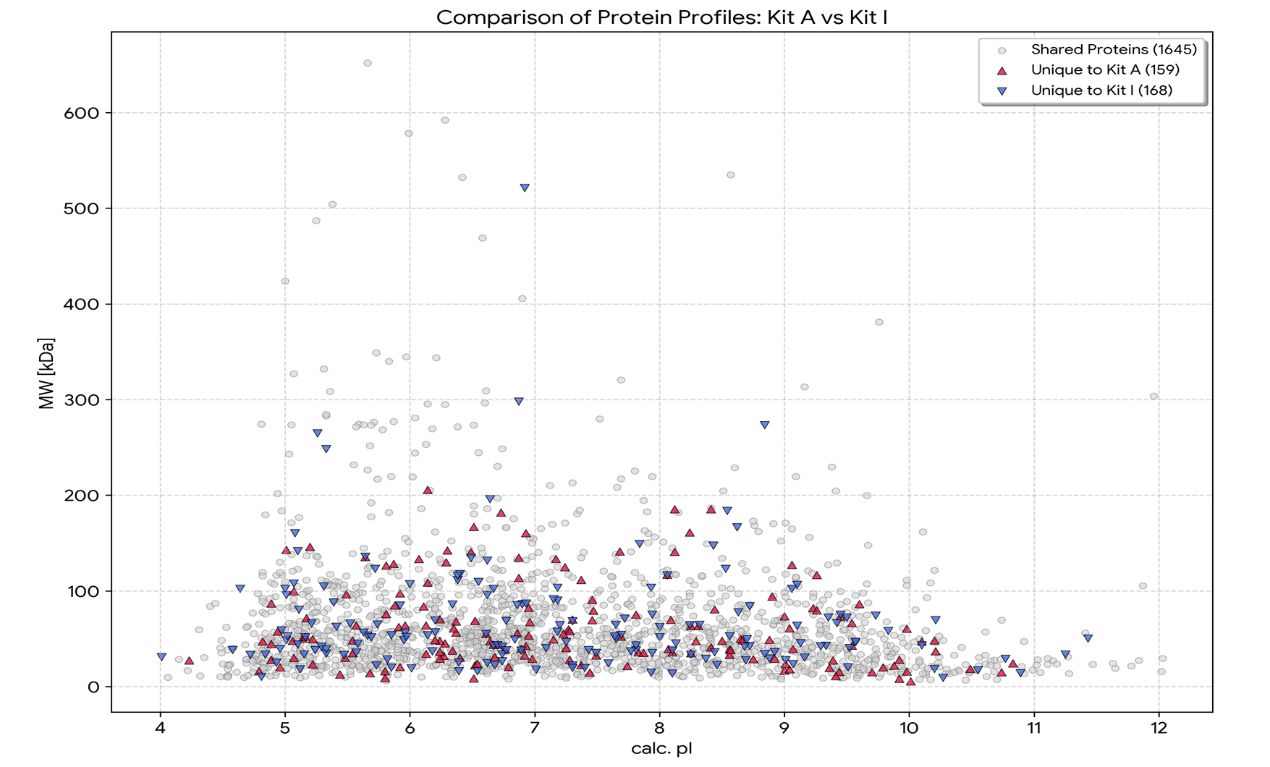
*

Figure S1 Scatter plot comparing differences in total enriched HCPs between kits A and I.
